# Abnormal auditory pathways in *PHOX2B* mutation positive congenital central hypoventilation syndrome

**DOI:** 10.1186/s12883-015-0299-z

**Published:** 2015-03-22

**Authors:** Ha Trang, Tarif Masri Zada, Fawzia Heraut

**Affiliations:** French Center of Reference for Central Hypoventilation, Robert Debré University Hospital, EA 7334 REMES, Paris-Diderot University, 48 boulevard Serurier, 75019 Paris, France; Department of Physiology, Robert Debré University Hospital, Paris, France; French Center of Reference for Central Hypoventilation, Robert Debré University Hospital, Paris, France

**Keywords:** Auditory evoked potentials, Central hypoventilation, Autonomic nervous system, *PHOX2B*, Congenital central hypoventilation syndrome, Central control of breathing

## Abstract

**Background:**

Congenital central hypoventilation syndrome (CCHS) is a rare disease characterized by severe central hypoventilation due to abnormal autonomic control of breathing. The *PHOX2B* gene, mutations of which define the disease, is expressed in a group of nuclei located in brainstem areas. Pathways controlling breathing and auditory pathways traverse very similar anatomic structures. In the present study, we measure brainstem auditory evoked potentials (BAEP) to assess auditory pathways in CCHS and investigate to which extent brainstem auditory pathways are also affected.

**Methods:**

BAEPs were measured in 15 patients with *PHOX2B* mutations positive CCHS (8 boys and 7 girls. mean age 6.3 yrs ± 5) as part of their regular follow-up in the Centre of reference for central hypoventilation (Robert Debré University Hospital. Paris. France).

**Results:**

BAEP responses were found normal in nine patients (60% of the study group) and abnormal in the other six (40%). Abnormal BAEPs which resulted from brainstem dysfunction were found in three patients (20%).

**Conclusion:**

Dysfunction of brainstem auditory pathways can be observed in CCHS. However, auditory evoked responses can be normal in the disease, therefore suggesting much more complex yet-to-be determined interactions between pathways and functions of central control of breathing and of control of hearing.

## Background

Congenital central hypoventilation syndrome (CCHS) is a rare disease characterized by abnormal autonomic control of breathing resulting in severe alveolar hypoventilation that is most marked during non-rapid eye movement sleep [1,2]. Incidence of CCHS is estimated to be around 1 for 200 0000 live births [3]. Mutations of the *PHOX2B* gene have been found in over 90% of the cases [4,5]. CCHS is frequently associated with a number of clinical conditions caused by abnormal development of the autonomic nervous system: Hirschsprung’s disease [5,6], neural crest tumors [3], reduced or absent central chemosensitivity and dyspnea sensations [7,8], heart rate and blood pressure dysregulation [9-11], esophageal dysmotility [12] and ocular disorders [13]. Widespread dysfunction of brainstem structures has been shown based upon functional magnetic resonance imaging (fMRI) studies of patients with CCHS [14-18].

Brainstem auditory evoked potentials (BAEP) are the changes in the ongoing electric activity generated in auditory pathways of the brain that is elicited by external auditory stimuli. Auditory pathways start from the auditory nerve (wave I) to the cochlear nuclei (wave II) in the medulla to the superior olivary complex (wave III) in the pons and end in the inferior colliculus (waves IV and V) in the midbrain. Prolonged latencies of the different waves and prolonged interpeaks latencies of BAEPs are considered as resulting from dysfunction of auditory pathways [19]. Recordings of BAEPs are used as a non-invasive neurophysiologic method to assess brainstem function, both in adults and in children, and may aid in the evaluation of various neurologic diseases.

Very limited BAEP data are available in CCHS. When studying 4 young toddlers with CCHS (age 6 weeks to 15 months), the authors found abnormal wave III and subsequently suggested a disruption in the auditory pathways at the level of mid to upper brainstem, an area close to that of central respiratory control [20]. Another patient with CCHS has been reported as having numerous alterations in the electronystagmograph and auditory potentials [21]. The genetic status of these patients was unknown, as these observations were reported long before identification of *PHOX2B* mutations as responsible of CCHS [4].

In the present study, we measured BAEPs in a large group of patients with *PHOX2B* mutations positive CCHS. We aimed to assess the integrity of auditory pathways in CCHS and investigate the extent in which brainstem auditory pathways were affected in the disease.

## Methods

### Patients

Fifteen children and adolescents with CCHS (8 boys and 7 girls. mean age 6.3 yrs ± 5) underwent BAEP recordings. The latter were part of a multidisciplinary assessment performed in the CCHS patients followed-up in the French Center of reference for central hypoventilation (Robert Debré University Hospital. Paris. France). Table 1 shows the main clinical characteristics of the study group. All patients had alveolar hypoventilation while spontaneously breathing during overnight polysomnography, absent respiratory response to exogenous CO_2_ and presence of mutations in the PHOX2B gene, all of these being +5 to +7 alanine expansions. Diagnosis was made in the first days of life for all, except for two of them (N°10 and 15). None was born prematurely. Hirschsprung’s disease was associated in three patients (N°3, 7 and 9). The study was approved by the Robert Debré Hospital Ethics Committee. Parents received full information before giving a written consent to undergo the procedure and publish individual clinical data. Parents were present throughout the procedure.

**Table 1:** **Main clinical characteristics of the study group**

**Patient n°**	**Age (years)**	**Sex**	**Onset of CCHS**	**Hypoventilation**	**Assisted ventilation**	**Hirschsprung**	**Neural crest tumour**
1	0.1	F	Neonatal	Sleep	Sleep	No	No
2	0.8	M	Neonatal	Sleep	Sleep	No	No
3	1	M	Neonatal	Sleep	Sleep	Yes	No
4	1.4	M	Neonatal	Sleep	Day O2 & night vent*	No	No
5	2.6	M	18 mo	Sleep	Sleep	No	No
6	3.8	M	Neonatal	Sleep	Sleep	No	No
7	4.1	F	Neonatal	Sleep	Sleep	No	No
8	5.6	F	Neonatal	Sleep	Sleep	No	No
9	6.2	M	Neonatal	Mild sleep hypoventilation	No	No	No
10	7.1	F	Neonatal	Sleep	Sleep	No	No
11	9.6	M	Neonatal	Sleep	Sleep	Yes	No
12	10.9	F	Neonatal	Sleep	Sleep	No	No
13	12	F	Neonatal	Sleep	Sleep	No	No
14	13	M	Neonatal	Sleep	Sleep	Yes	No
15	17	F	3 mo	Sleep	Sleep	No	No

*Patient n°4 had associated bronchopulmonary dysplasia.

### Methods

Without any previous sedation, each patient underwent BAEPs using a Nicolet Spirit system. BAEPs were recorded by surface electrodes attached by tape to the scalp at the Cz area and at the ipsilateral earlobe after 2000 monaural stimulations of 70 dB intensity and with a controlateral “masking” of 40 dB intensity. If no response is obtained with 70 dB stimulation intensity, a new stimulation is done with 90 dB intensity. The objective auditory level of the V wave is systematically studied by progressively decrease of the stimulation intensity. Latencies and amplitudes of the I, II, III, IV and V waves were measured and the interpeaks latencies (IPL) of waves III-I. V-I and V-III were analyzed. Data from patients were compared to those obtained in our laboratory from 30 healthy control matched subjects in different age groups (0-2 months. 3-5 months. 6-18 months. 1.5-5 years. 6-10 years and young adults). Table 2 displays the upper values considered for defining abnormalities, expressed as mean + 2.5 SD for each age group.

**Table 2:** **BAEPs obtained in 30 control subjects in different age groups**

	**LEFT EAR**						**RIGHT EAR**					
**AGE GROUP**	**0-2months**	**3-5months**	**6-18months**	**1.5-5years**	**6-10years**	**young adults**	**0-2months**	**3-5months**	**6-18months**	**1.5-5years**	**6-10years**	**young adults**
LATENCIES												
wave I	1.7	1.6	1.6	1.6	1.6	1.5	1.7	1.6	1.6	1.6	1.6	1.5
wave II	3.1	2.8	2.8	2.7	2.7	2.7	3.0	2.9	2.8	2.7	2.7	2.7
wave III	4.6	4.3	4.0	3.8	3.8	3.7	4.6	4.2	4.0	3.7	3.7	3.8
wave IV	5.7	5.3	5.1	4.9	4.8	4.9	5.5	5.2	5.0	4.8	4.8	4.9
wave V	6.8	6.4	6.1	5.6	5.6	5.4	6.6	6.4	5.9	5.7	5.6	5.5
IPL III-I	2.9	2.7	2.4	2.2	2.2	2.2	2.9	2.6	2.3	2.2	2.1	2.2
IPL V-III	2.2	2.1	2.0	1.8	1.8	1.7	2.1	2.2	2.0	1.9	1.8	1.7
IPL V-I	5.1	4.8	4.5	4.1	4.1	3.9	4.9	4.8	4.3	4.1	4.0	3.9

Data are expressed as the upper values for defining abnormalities (=mean + 2 SD) for each age group. IPL, interpeaks latencies (units in msec).

### Statistical analysis

Comparison of right and left BAEP data was done with the paired two-tailed Student’s *t*-test (SPSS version 19, IBM SPSS inc). Difference was considered significant for P values of 0.05 or less.

## Results

Table 3 shows BAEP responses for the study group. Normal BAEP responses were observed in nine children (60%) and abnormal responses in the other six (40%). For three patients (N°6, 11 and 14), abnormalities consisted in significantly delayed responses of the auditory nerve (wave I) with normal inter-latencies IPL V-I and V-III in patients 6 and 11 but asymmetric responses in patient 14. These results were considered as peripheral auditory transmission dysfunction. For two other patients (N°3 and 4), latencies of the three first waves (I, II, III) were normal but significantly delayed latencies or lost responses of the fourth and fifth waves responses were observed with increased or incalculable IPL V-I and V-III. These results were considered as brainstem dysfunction. Finally, one patient (N°2) showed asymmetric and drastically altered responses with an unidentified bilateral wave II suggesting major peripheral auditory dysfunction and loss or prolonged latencies of IV and V waves with loss or increased interpeaks V-I and V-III suggesting brainstem dysfunction.

**Table 3:** **BAEPs responses for the whole group (units in msec)**

	***L A T E N C I E S***										***I N T E R P E A K S L A T E N C I E S***					
**Patient n°**	**I**		**II**		**III**		**IV**		**V**		**III-I**		**V-III**		**V-I**	
	**R**	**L**	**R**	**L**	**R**	**L**	**R**	**L**	**R**	**L**	**R**	**L**	**R**	**L**	**R**	**L**
1	1.7	1.6	3	3	4.4	4.5	5.8	5.7	6.6	6.5	2.7	2.9	2.2	2	4.9	4.9
2* #	2.3	1.8	/	/	4.7	/	/	/	/	7.2	2.4	/	/	/	/	5.4
3*	2.1	2.2	3	4.7	4.1	6.5	5.4	/	6.3	/	2	4.3	2.2	/	4.2	/
4*	2.2	2	3.3	/	4.6	4.4	/	/	6.6	6.6	2.4	2.4	2	2.2	4.4	4.6
5	1.8	1.8	2.6	2.7	3.9	3.8	5.1	5.3	5.8	5.9	2.1	2	1.9	2.1	4	4.1
6 #	2.3	2.5	3.2	3.5	4.5	4.6	5.9	5.8	6.3	6.4	2.2	2.1	1.8	1.8	4	3.9
7	2.2	2.2	3	3.4	4.2	4.4	5.2	5.6	6.3	6.3	2	2.2	2.1	1.9	4.1	4.1
8	1.4	1.3	2.7	2.4	3.6	3.4	4.9	4.6	5.7	5.3	2.2	2.1	2.1	1.9	4.3	4
9	1.6	1.7	2.7	2.8	3.9	4	4.8	5.1	5.7	5.9	2.3	2.3	1.8	1.9	4.1	4.2
10	1.9	2	2.6	2.7	3.7	3.8	4.7	4.7	5.7	5.7	1.8	1.8	2	1.9	3.8	3.7
11 #	2.5	2.3	/	/	4.1	4.2	4.9	5.4	6.1	6.1	1.6	1.9	2	1.9	3.6	3.8
12	1.7	1.8	2.9	2.8	3.9	3.9	5.3	5.2	5.8	5.9	2.2	2.1	1.9	2	4.1	4.1
13	1.7	1.6	2.8	2.8	3.8	3.8	5.2	5.1	5.8	5.7	2.1	2.2	2	1.9	4.1	4.1
14 #	2.5	1.9	/	3	/	4.1	/	5.8	/	6.2	/	2.2	/	2.1	/	4.3
15	1.6	1.7	2.9	2.7	3.8	3.8	5	5	5.9	5.8	2.2	2.1	2.1	2	4.3	4.1

Abnormal BAEPs were found in 6 patients. *Brainstem dysfunction. # Peripheral auditory dysfunction.

For the whole group, mean values of wave latencies and interpeaks latencies did not differ between right and left ears of the patients. BAEPs data were not correlated with presence of associated clinical conditions or with genetic status.

## Discussion

Our study indicates abnormalities of the BAEPs in 40% of the patients with CCHS examined. However, abnormalities related to brainstem dysfunction were observed in 20% of them only. Significant alterations in the brainstem autonomic structures in CCHS have been described using fMRI studies [14-18]. The alterations are thought to be caused by maldevelopment or neural demyelization as a consequence of gene mutations. This likely accounted for the delayed BAEPs observed in some of our patients.

Several authors have reported that BAEP responses in neonates and preterm infants are affected by the maturity of the auditory system. However, the effect of maturity is more evident in premature infants; thus, the response pattern in these children may differ from those in term neonates [22-24]. In our group, we had no premature infant. We also compared patients’ data to those obtained from healthy control matched subjects corrected for age to determine abnormal BAEP. Nevertheless, alterations in BAEPs found in our CCHS patients were not correlated with age, i.e. patient 1 of 1 month of age had normal data, whereas patient 14, aged 13 years showed altered responses.

One new finding of the present study was dysfunction of peripheral auditory transmission in some patients without any auditory infection such as otitis or rhinopharyngitis. One may hypothesize a cochlear dysfunction secondary to a possible cochlear malformation.

More interestingly, the normal conduction in BAEPs found in the majority of our patients showed the complexity of the roles of the anatomic structures in this disease. In fMRI studies conducted in patients with CCHS, abnormalities appeared in brainstem areas involving the ventral midbrain, pons, and dorsal and ventral medulla. These areas are principally traversed by fibers of significance for breathing and autonomic control, but not by fibers for auditory control that are situated more laterally. The auditory fibers enter the brainstem at the lateral side of the pons lateral to the restiform body and immediately split up to enter the dorsal and ventral cochlear nuclei. The auditory pathways to the cortex are complex. The further cell stations are the superior olive, the nucleus of the lateral lemniscus, the inferior colliculus on the roof of the midbrain and the medial geniculate body. Between cochlear nuclei and medial geniculate body, there are at least two neurons. Many but not all fibers decussate. Of these decussations, the most ventral one at the level of the superior olive is the strongest, known as the trapezoid body. The pathway between superior olive and inferior colliculus is a compact bundle of fibers known as the lateral lemniscus. The distribution of the different waves recorded through auditory evoked potentials indicate wave I as the volley of eight nerve action potentials generated by the click stimulus in the segment of the nerve close to the cochlea. Waves II and III are assigned to the cochlear nucleus and superior olivary complex, respectively. Waves IV and V are considered generated in inferior colliculus or lateral lemniscus [19]. Therefore, we could suggest that BAEP abnormalities may be observed in patients with CCHS when damage or underdevelopment is more diffuse in brainstem areas.

## Conclusions

Using BAEP studies, we found arguments for dysfunction of brainstem auditory pathways in a few patients with CCHS. In the others, BAEPs were normal. This finding likely suggests much more complex yet-to-be determined interactions between pathways and processes of central control of breathing and of control of hearing.

## Abbreviations

BAEP: Brainstem auditory evoked potentials
CCHS: Congenital central hypoventilation syndrome
fMRI: Functional magnetic resonance imaging
